# Diagnostic coding of chronic physical conditions in Irish general practice

**DOI:** 10.1007/s11845-021-02748-3

**Published:** 2021-09-02

**Authors:** Ivana Pericin, James Larkin, Claire Collins

**Affiliations:** Irish College of General Practitioners, 4–5 Lincoln Place, Dublin, Ireland

**Keywords:** Chronic disease, Diagnostic coding, General practice, ICD10, ICPC2

## Abstract

**Background:**

Chronic conditions are responsible for significant mortality and morbidity among the population in Ireland. It is estimated that almost one million people are affected by one of the four main categories of chronic disease (cardiovascular disease, chronic obstructive pulmonary disease, asthma, and diabetes). Primary healthcare is an essential cornerstone for individuals, families, and the community and, as such, should play a central role in all aspects of chronic disease management.

**Aim:**

The aim of the project was to examine the extent of chronic disease coding of four chronic physical conditions in the general practice setting.

**Methods:**

The design was a descriptive cross-sectional study with anonymous retrospective data extracted from practices.

**Results:**

Overall, 8.8% of the adult population in the six participating practices were coded with at least one chronic condition. Only 0.7% of adult patients were coded with asthma, 0.3% with COPD, 3% with diabetes, and 3.3% with CVD. Male patients who visited their GP in the last year were more likely to be coded with any of the four chronic diseases in comparison with female patients. A significant relationship between gender and being coded with diabetes and CVD was found.

**Conclusions:**

For a likely multitude of reasons, diagnostic coding in Irish general practice clinics in this study is low and insufficient for an accurate estimation of chronic disease prevalence. Monitoring of information provided through diagnostic coding is important for patients’ care and safety, and therefore appropriate training and reimbursement for these services is essential.

## Background

Chronic diseases are one of the major health challenges in the twenty-first century [1–3]. Due to their slow progression and long period of treatment, they produce negative effects on an individual’s physical, emotional, and mental wellbeing, frequently leading to ill health and mortality [4, 5]. The population of Ireland is ageing rapidly, with a high number of people older than 50 suffering from multimorbidity [6–8]. The prevalence of chronic conditions among the adult population in Ireland is increasing [9, 10]. The prevalence of the four main categories of chronic diseases among adults in Ireland has been estimated to be 9.4% for asthma [11], 9.8% for COPD [11], 5.2% for diabetes [12], and 4.3% for CVD [13]. In Ireland, 80% of all patients who visit a general practitioner (GP) have a chronic disease [14]. Hence, general practice represents a great source of information about the health of the Irish population.

In the last 20 years, practice management software systems became an integral part of Irish general practice [15]. As complex healthcare information systems, they provide assistance in managing and recording clinical data including patients’ demographic information, diagnosis, prescriptions, etc. [15]. Healthcare systems also facilitate diagnostic coding in general practice, which is important for systematic classification of clinical information and data collection for research and clinical audits [16]. Regarding chronic diseases, regular coding of diagnosis contributes to better patient’s care and improves health management and prevention strategies on the population levels [17]. Although the recording of clinical data in a systematic and accurate form is highly beneficial for GPs regarding patient care and successful management of chronic diseases [18, 19], there are barriers which impact clinical recording. Previous research highlighted time constraints, level of motivation, and limitations of the coding systems and terminologies [18] as some of the obstacles that healthcare professionals commonly face. As a consequence, an absence of recording of diagnosis, as well as discrepancies between diagnoses and prescribed medications, often occur, and could significantly impact the quality of patient care [20]. One of the solutions suggested by previous studies was that an introduction of financial incentives could significantly improve clinical coding and recording of patients’ data, including diagnosis, risk factors, and prescriptions [21–23]. Financial incentives already exist in a number of countries, including the UK, Germany, France, Netherlands, and Australia [16, 24–26].

Since January 2020, a chronic disease management (CDM) programme is in operation in Ireland for patients aged over 75 years holding a general medical service (GMS) card or a GP visit only (GPVC) where GPs are reimbursed for the provision of specified care for patients with type 2 diabetes, asthma, COPD, and CVD [27]. However, a national structured diabetes programme has existed since 2015 [28] and a structured secondary prevention programme for cardiovascular disease for approximately 20% of GPs since 2002 [29]. It is intended that the new CDM programme will be expanded to other age groups on a phased basis. Data from this programme are currently returned to the Health Services Executive (HSE) via an electronic portal, however software tools for GPs to appraise their own practice’s clinical management, including those that are related to their CDM activity, are not currently available. Although there is an overall agreement on the importance of diagnostic coding as part of general practice data management, technical issues, as well as overall reluctancy to code (due to time constraints and concerns regarding the accuracy of the diagnostic codes), prevent the establishment of global diagnostic coding in Ireland [30]. As a result of the implementation of the Irish government’s Sláintecare strategy, the future management of chronic disease will rely more heavily on general practice [31]. The planning for such requires a clear picture of the extent of coding in Irish general practice—thus this paper aims to contribute to the knowledge base relating to this aspect.

## Methods

The data presented here were collected as part of a multi-phase research project ‘Making Every Consultation Count’ (MECC) in Irish general practice. The MECC project took place in the period from October 2016 to April 2019, with an aim to investigate the feasibility of recording chronic disease risk factors and delivering appropriate brief interventions for all adult patients, in the general practice setting.

This paper reports on one aspect of this project—a descriptive cross-sectional analysis of practice data based on electronic medical records retrieved retrospectively from practices using the Socrates patient management software, from two midland regions in Ireland.

Following ethical approval for the overall project, a letter was sent to all GP practices in the two areas outlining the nature of the study and the criteria for participation. These areas were included on the basis of the funding criteria and having a GP champion for the project and health system supports for the wider interventional project in these areas. The initial letter was followed by a presentation where researchers provided more detailed information of the study to all GPs who were interested in participating. All of the interested GPs were provided with an information sheet and consent form prior to their participation in the study. A total of 21 practices expressed an initial interest and attended the information evening. Thirteen practices subsequently participated in the study. A research grant of €1500 was given to all participating practices to cover research-related expenses. Data from the six practices utilising the Socrates practice management software system is included in this paper as data extraction was not reliable or possible from the other systems.

In order to retrieve the data from the participating practices, an uploader was developed in cooperation with the software provider. The uploader allowed the extraction of data including: patients’ demographics data, consultation frequency in the past year, codes of chronic diseases (ICPC2 and ICD 10 international classifications), and the date when a chronic disease was first coded. All the data retrieved was anonymous, extracted retrospectively at practice level, and uploaded to a central database via a secure connection.

The practice population was defined as all adult active patients. All patients who are registered or visited the practice and therefore are entered on the PMS system are by default classified as *active*; in order for their status to differ from this, they must be actively reclassified as inactive, deceased, or archived.

Data on ever coding of chronic disease was extracted for all active adult patients and for adult patients who had attended the practice at least once in the past year.

The main focus of this paper is on four main physical health chronic diseases, namely asthma, diabetes, CVD, and COPD. The ICPC2 and ICD 10 codes relevant to these categories can be found in Table 1.

**Table 1:** Relevant disease codes for four main categories of chronic physical diseases

Chronic condition	Disease codes	
	ICPC2	ICD10
Asthma	R96	J45, J45.0, J45.1, J45.8, J45.9, J46
Diabetes	T89, T90, W85	E10, E11, E12, E13, E14, O24, O24.4, O24.9
Cardiovascular	K22, K71, K73, K74, K76, K77, K78, K79, K80, K81, K82, K83, K84, K89, K90, K91, K92, K93, K94, K99	I24.8, I24.9, G46.3, G46.4, I20, I21, I25, Z82.3, Z82.4, Z86.7, Q20, Q21, Q22, Q23, Q24, Q25, Q26, Q27, Q28, Q29, R01.1, R03.0, G45, I60, I61, I62, I63, I64, I65, I66, I67, I68, I69, I70, I80, I05, I06, I07, I08, I09, I10, I11, I12, I13, I14, I15, I16, I26, I27, I28, I30, I31, I32, I33, I34, I35, I36, I37, I38, I39, I40, I41, I42, I43, I44, I45, I46, I47, I48, I49, I50, I51, I52, I81, I82, I83, I84, I85, I86, I87, I71, I72, I73, I74, I75, I76, I77, I78, I79, I95, I96, I97, I98, I99
COPD	R95	J40, J41, J42, J43, J44

The SPSS Statistics package version 23 was used to analyse the data, using frequencies and descriptive statistics to summarise demographic information, consultation rates, and the frequency of coding of a particular chronic disease category.

## Results

### Overall description of the practices

The study was conducted with six practices which utilised the Socrates software system. Across all six practices, the total number of GPs was 13, where 11 GPs worked full-time and two GPs part-time.

Data was available for 13,572 active adult patients who were recorded as having visited their GP practice at least once in the last year. The range of patient visits in all instances is wide. The median number of consultations in the last year was substantially higher for GMS patients (median = 11) in comparison with private patients (median = 4) (Table 2). Female patients had more consultations in the last year (median = 7) in comparison to male patients (median = 6).

**Table 2:** Description of the participating practices

	GPs in the practice	Adult patients who had at least one consultation in the last year (*n*)	Number of consultations in the last year (for adult patients) Median/range		
			Total	GMS	Private
Practice 1	1.5	1191	7 (0–72)	13 (0–73)	4 (0–35)
Practice 2	2	2391	8 (0–77)	13 (0–77)	5 (0–73)
Practice 3	1.5	2620	4 (0–75)	8 (0–75)	2 (0–47)
Practice 4	2	2612	6 (0–100)	11 (0–100)	3 (0–63)
Practice 5	1	1083	8 (0–79)	10 (0–79)	4 (0–25)
Practice 6	4	3675	7 (0–75)	9 (0–75)	4 (0–49)
Total	13	13,572	7 (0–100)	11 (0–100)	4 (0–73)

**Fig. 1:**
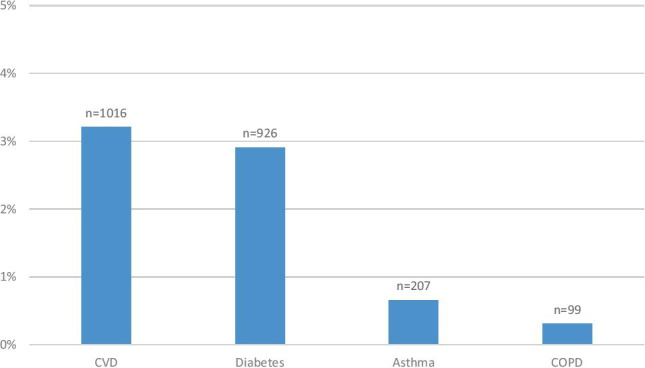
Percentages of adult population coded with four main categories of chronic disease

In total, 8.8% (*n* = 2788) of all active adult patients were coded with at least one chronic disease in their lifetime. Among these patients, CVD and diabetes were found to be the most commonly coded, accounting for 3.2% (*n* = 1016) and 2.9% (*n* = 926) of all adults, respectively. In total, less than 1% of patients were coded with asthma (0. 7%, *n* = 207) or COPD (0.3%, *n* = 99) (Fig. 1).

Looking in detail at patients who attended the practice in the past year, we see that 19.4% (*n* = 2635) were ever coded with a chronic condition. Among these patients, 7.2% were ever coded with CVD; 6.5% with diabetes; 1.4% with asthma, and 0.7% with COPD.

While examining the demographics of this sample, it was evident that the proportion of patients coded with CVD, diabetes, and COPD sharply increases with age, as would be expected (Table 3). The same was not true for asthma where the proportion diagnosed did not increase with age.

**Table 3:** Demographics of the patients who had at least one consultation in the preceding year and proportion-coded with each of the chronic diseases

	All active adults who visited the practice in the last year % (*n*)	Proportion of patents within each demographic group ever coded with the condition listed			
		**Diabetes** % (*n*)	**Asthma** % (*n*)	**COPD** % (*n*)	**CVD** % (*n*)
Gender*					
Male	47.5 (6445)	8.4 (540)	1.7 (110)	0.8 (54)	9.4 (603)
Female	52.5 (7115)	5.4 (385)	1.4 (97)	0.6 (45)	5.8 (413)
Age					
< 24	8.7 (1186)	0.8 (10)	1.43 (19)	0 (0)	0.2 (2)
25–44	32.8 (4454)	1.4 (70)	1.5 (77)	0 (0)	1.0 (46)
45–64	32.6 (4421)	6.3 (289)	1.2 (59)	0.6 (28)	5.9 (265)
> 65	25.9 (3511)	15.7 (557)	1.3 (52)	1.9 (71)	19.1 (703)
Patient status					
GMS	48.6 (6587)	10.2 (671)	1.7 (114)	1.2 (79)	10.7 (707)
Private	46.8 (6352)	3.2 (204)	1.3 (84)	0.3 (16)	3.9 (249)
DVC	4.0 (542)	9.0 (49)	8.9 (9)	0.7 (4)	10.7 (58)
Other	0.6 (82)	1.2 (1)	0 (0)	0 (0)	2.4 (2)
Number of consultations in the last year	Median: 7 (IQR: 10)	Median: 15 (IQR: 16)	Median: 10 (IQR: 13)	Median: 19 (IQR: 18)	Median: 14 (IQR: 14)

^*^Gender was not recorded or ‘unspecified’ for 12 patients

Regarding the patients’ status, the majority of patients who were coded with any of the four main categories of chronic diseases were holders of the GMS card.

The median number of consultations in the past year is greater for those with each of the chronic conditions listed compared to patients not coded with any of these conditions. The median number of annual consultations for all adult patients who attended the practice in the past year was seven. The median was five for patients never coded with a chronic condition; 13 for those ever coded with any chronic condition and 13 for those coded with any of the four physical conditions considered here.

A chi-square test revealed a statistically significant relationship between gender and being coded with CVD or diabetes. Male patients were more likely to be coded with CVD (*χ*^2^ (1 df) = 5 9.203, *P* = 0.000) and diabetes (*χ*^2^ (1 df) = 43.737, *P* = 0.000) in comparison with female patients.

## Discussion

Diagnostic coding in Irish general practice was found to be very low. Overall, 8.8% of the adult population was coded with at least one chronic condition, which is considerably lower in comparison with other countries, such as the UK and Australia, where more than 30% of the adult population has a chronic disease [32, 33]. The health statistics based on data retrieved from general practice in these countries are often used to inform health policies. Inaccurate data produces a harmful effect on adequate allocation and management of resources for the prevention and treatment of chronic diseases. The consistency of coding also impacts the availability of a reliable epidemiological database and the ability of practices to undertake an audit and to participate in clinical trials [34]. The Australian government encourages general practices to electronically record all diagnoses [25]. There is an absence of government initiatives or policies in Ireland which could encourage consistent recording of diagnoses across all patient groups. Thus far, the Sláintecare Implementation Strategy has highlighted that a key role of the management of chronic diseases will be held by general practice [31]. Considering that a vast majority of the GP consultations are due to chronic conditions, the incentivisation of the diagnostic coding appears to be an important step in order to ensure an efficient and effective chronic disease management. It may also enhance healthcare planning which is essential for overall patient care.

When comparing the estimates of the prevalence of chronic conditions among the adult population in Ireland, where 9.4% of adults are diagnosed with asthma [11] and 9.8% with COPD [11], the present study found that only 0.7% of adult patients were coded with asthma and 0.3% with COPD. Regarding diabetes, 5.2% of adults were estimated to be diagnosed with this condition [12], but only 3% were coded with it. For the patients with CVD, a total of 3.2% of patients were coded with CVD, in comparison with 4.3% of patients diagnosed with CVD [13]. Although under-coding takes place in all four main categories of chronic disease, higher levels of coding were evident for patients who were diabetics or who had CVD. Some of the previous research found that coding of diabetes tends to be of higher quality than coding for asthma, due to clearer diagnostic features [35]. However, in the case of Ireland, it could be that the existence of structured programmes for diabetic care and CVD for a far longer period with financial reimbursement [28, 29] could be considered a ‘push’ factor for GPs, to code these conditions more regularly. In 2015, the Diabetes Cycle of Care programme was introduced nationally by the HSE [28]. The programme represents the Irish government’s initiative to reimburse GPs for every registered diabetic patient as well as to provide two structured visits each year for patients with type 2 diabetes [28]. The initiative was established in order to improve the overall care for type 2 diabetic patients in primary care while reducing the capacity constraints for these patients within the specialist diabetic clinics. In the first year of launch, almost 85,000 patients were registered for the scheme, accounting for more than 11 million in payments to GPs [36]. Furthermore, the Heartwatch programme was established in 2003 across GP practices in Ireland and represents a GP scheme focused on secondary prevention for cardiovascular diseases, delivered within primary care [29, 37]. In this programme, GPs see patients four times per year and are reimbursed per visit per patient. Approximately 20% of practices nationally participate in the Heartwatch and about 25,000 patients are registered on the programme annually [37]. Participation in both programmes requires that patients are coded with the relevant condition and demonstrates that despite time constraints and additional workloads, the diagnosis could be efficiently recorded once incentivised. A new Chronic Disease Management (CDM) programme was introduced in January 2020 covering only patients aged 75 years and older and eligible for free GP care [27]. The CDM has resourced GPs to manage eight chronic conditions and will each year incorporate a younger cohort of patients, as well as  providing an incentive to GPs to code their patients living with these conditions. This is likely to increase coding for these conditions. The study presented in this paper took place in the period between 2016 and 2019 prior to the introduction of the CDM programme when GPs were not incentivised to code all the conditions required by the programme.

Regarding gender, male patients who visited in the last year were more likely to be coded with any of the four main categories of chronic diseases in comparison with female patients. This is especially evident for diabetes and CVD, where a significant relationship between gender and being coded with diabetes and CVD was found. A systematic review and meta-analysis conducted in Ireland highlighted that the prevalence of doctor-diagnosed diabetes among the male population was higher in comparison with female [12]. This finding is consistent with international literature, where globally men develop diabetes at a higher rate than their female counterparts [38–40]. In 2019, 17.2 million more men than women were affected by this disease [38]. With regard to CVD, the European Heart Network estimated that in 2015, 49 million people were living with CVD in the EU, of which 52% were women [41]. However, with a focus on Ireland, the same report outlined that a number of males affected by CVD were slightly higher (*n* = 142,518) in comparison with females (*n* = 126,358), which was consistent with our findings.

When comparing with other countries including the UK, Netherlands, and Australia [30], where data retrieved from general practice represents a rich source of information, in Ireland due to the low level of diagnostic coding, the quality of data collected remains poor [34, 42]. Previous research demonstrated that additional extensive training in computer use and coding principles of practice staff could contribute to a better quality of coding [12, 42]. Currently in Ireland, there is no formal training of GPs regarding computer use or diagnostic coding. Therefore, proficiency in this area depends on the motivation of individual GPs and priorities towards coding which are established on an individual practice level. The limitations of some of the existing practice management systems and coding schemas may also contribute  to lower than desired coding levels [18, 34].

### Strengths and limitations

The results of the study have identified significantly low levels of diagnostic coding of chronic diseases and could serve to bolster the development of future health policies with more focus on the improvement of the validity of diagnostic coding in general practice.

The study sample consisted of six general practices, which limits its representativeness. Originally, we intended to include a larger number of general practices; however, difficulties were experienced uploading data from some of the practice management software systems. Therefore, only practices from one PMS system managed to upload their data and were included in the study. The impact of this on coding rates within practices is unknown.

Self-selection bias could also be applicable to our study since there is a possibility that the participants may have had a special interest in the topic and therefore might be more likely than the average GP to recognise, diagnose, and code chronic diseases.

## Conclusion

Persistent under-coding leads to an underestimation of the burden of chronic diseases. Improving the validity of diagnostic coding should be a priority in order to provide more accurate prevalence and impact data. Training in data recording and coding principles for practice staff is required. Reimbursement and training for general practices to regularly and accurately maintain patients’ electronic records would contribute to adequate monitoring of chronic conditions and better identification of at-risk groups. However, data recording should not become an end in itself but a means to monitor patient-related factors and the impact of interventions. The reporting functions in GP practice management software systems are insufficient for research and audit purposes—this needs to be considered when designing national IT infrastructure for the health system. As new chronic disease programmes are developed, the data requirements will change, and the PMS systems used by GPs must be able to accommodate these.

## Data Availability

Data are available on reasonable request to the corresponding author.
